# Development of Bioactive Cotton, Wool, and Silk Fabrics Functionalized with *Origanum vulgare* L. for Healthcare and Medical Applications: An In Vivo Study

**DOI:** 10.3390/pharmaceutics17070856

**Published:** 2025-06-30

**Authors:** Aleksandra Ivanovska, Anica Petrović, Tamara Lazarević-Pašti, Tatjana Ilic-Tomic, Katarina Dimić-Mišić, Jelena Lađarević, Jovana Bradić

**Affiliations:** 1 Innovation Center of the Faculty of Technology and Metallurgy, University of Belgrade, Karnegijeva 4, 11000 Belgrade, Serbia; 2 Department of Pharmacy, Faculty of Medical Sciences, University of Kragujevac, Svetozara Makovića 69, 34000 Kragujevac, Serbia; anica.petrovic@fmn.kg.ac.rs (A.P.); jovana.bradic@fmn.kg.ac.rs (J.B.); 3 Center of Excellence for Redox Balance Research in Cardiovascular and Metabolic Disorders, Svetozara Makovića 69, 34000 Kragujevac, Serbia; 4 VINČA Institute of Nuclear Sciences–National Institute of the Republic of Serbia, University of Belgrade, Mike Petrovica Alasa 12-14, 11000 Belgrade, Serbia; lazarevictlj@yahoo.com; 5 Institute of Molecular Genetics and Genetic Engineering, University of Belgrade, Vojvode Stepe 444a, 11042 Belgrade, Serbia; tatjana.ilic-tomic@imgge.bg.ac.rs; 6 Department of Chemical and Metallurgical Engineering, School of Chemical Engineering, Aalto University, 02150 Espoo, Finland; katarina.dimic-misic@aalto.fi; 7 Institute of General and Physical Chemistry, Studentski Trg 12/V, 11158 Belgrade, Serbia; 8 Faculty of Technology and Metallurgy, University of Belgrade, Karnegijeva 4, 11000 Belgrade, Serbia; jmirkovic@tmf.bg.ac.rs

**Keywords:** bioactive fabric, *Origanum vulgare* L., extract, antioxidant activity, antibacterial activity, release of extract bioactive compounds, healthcare application, medical textiles, wound dressing, in vivo

## Abstract

**Background:** This study presents an innovative approach to developing bioactive natural fabrics for healthcare and medical applications. **Methods:** An ethanol extract of *Origanum vulgare* L. (in further text: OE), exhibiting exceptional antioxidant (100%) and antibacterial activity (>99% against *E.coli* and *S.aureus*), was employed to biofunctionalize cotton, wool, and silk fabrics. **Results:** All biofunctionalized fabrics demonstrated strong antioxidant activity (>99%), while antibacterial efficacy varied by fabric: cotton > 54%, wool > 99%, and silk > 89%. OE-biofunctionalized wool possessed the highest release of OE’s bioactive compounds, followed by silk and cotton, indicating substrate-dependent release behavior. This tunable fabrics’ OE release profile, along with their unique bioactivity, supports targeted applications: OE-functionalized silk for luxury or prolonged therapeutic use (skin-care textiles, post-surgical dressings, anti-aging products), cotton for disposable or short-term use (protective wipes, minor wound coverings), and wool for wound dressings. The biocompatibility and cytotoxicity of OE-biofunctionalized wool were evaluated via in vitro assays using healthy human keratinocytes and in vivo testing in *Wistar albino* male rats. The obtained results revealed that OE-functionalized wool significantly accelerated wound closure (97.8% by day 14), enhanced collagen synthesis (6.92 µg/mg hydroxyproline), and improved tissue and systemic antioxidant defense while reducing oxidative stress markers in skin and blood samples of rats treated with OE-biofunctionalized wool. **Conclusions:** OE-biofunctionalized wool demonstrates strong potential as an advanced natural solution for managing chronic wounds. Further clinical validation is recommended to confirm its performance in real-world healthcare settings. This work introduces an entirely new application of OE in textile biofunctionalization, offering alternatives for healthcare and medical textiles.

## 1. Introduction

*Origanum vulgare* L., commonly known as oregano, is a perennial aromatic herb belonging to the Lamiaceae family and is widely recognized for its multifaceted uses [1]. Indigenous to various ecological regions and well-adapted to arid, rocky, and calcareous soils, particularly in the Mediterranean, Irano-Turanian, and Euro-Siberian zones, oregano is now extensively distributed across Europe, North America, Africa, and Asia [2,3]. Beyond its central role as a culinary spice in Mediterranean and Middle Eastern cuisines, oregano holds a longstanding place in traditional medicine, especially within European ethnobotanical practices [4]. Chemically, *Origanum vulgare* L. is characterized by a rich profile of secondary metabolites, including volatile oils (such as thymol and carvacrol), as well as phenolic acids like rosmarinic acid, terpenoids, and flavonoids [3]. These bioactive compounds provide the plant with a broad spectrum of pharmacological properties, including antibacterial, antioxidant, and anti-inflammatory, positioning *Origanum vulgare* L. as one of the most biologically potent species within the Lamiaceae family [5]. Such properties have spurred growing scientific interest and widespread commercial applications of oregano and its derivatives. For instance, oregano essential oils have been extensively studied for use in clinical therapies targeting various chronic conditions [6,7]; however, their broader application remains constrained by issues such as poor chemical stability [8]. In addition, oregano extracts have found valuable roles in pharmacology, cosmetology, aromatherapy, and agriculture [4,9,10]. Despite this extensive research and utilization, the potential utilization of *Origanum vulgare* L. extract for fiber biofunctionalization remains entirely unexplored.

In this context, the present study aims to go beyond the current state-of-the-art by addressing a critical knowledge gap through the first-time investigation of the biofunctionalization of cotton, wool, and silk fabrics using *Origanum vulgare* L. ethanol extract (in further text: OE). This novel approach paves the way for the development of bioactive natural textiles tailored for healthcare and medical applications. Following a simple and efficient extraction procedure, the chemical composition of the OE was assessed, and its antioxidant and antibacterial activities were evaluated to confirm its bioactivity. The bioactive extract was then employed for the fabric functionalization, the bioactivity of the OE-biofunctionalized fabrics was validated, and their ability to release key bioactive compounds into a skin-simulating physiological fluid was assessed. The results demonstrate that each type of fabric exhibits a unique bioactive profile and release behavior, which can be specifically tailored to distinct healthcare and medical needs. For instance, OE-biofunctionalized silk demonstrated excellent potential for luxury or prolonged skin-contact applications, such as post-surgical garments and anti-aging textiles. OE-biofunctionalized cotton was identified as suitable for short-term or disposable uses, including protective wipes and minor wound coverings. Meanwhile, OE-biofunctionalized wool showed promise as a material for wound dressings.

It is important to emphasize that this study distinguishes itself from conventional research, which often assumes the potential applicability of bioactive fabrics in healthcare and medical textiles without experimental validation. In contrast, this work assesses the cytotoxicity and therapeutic potential of OE-biofunctionalized wool fabric through a combination of in vitro and in vivo evaluations. Specifically, the cytotoxicity of OE-biofunctionalized wool was examined using healthy human keratinocyte cultures, while its wound healing efficacy was investigated in vivo employing full-thickness excision wound model in rats because it closely mimics acute clinical wounds and allows detailed evaluation of the wound healing process. Throughout the 14-day study protocol, wound healing progression was systematically monitored, along with biochemical quantification of hydroxyproline and oxidative stress markers in skin tissue, as well as the assessment of systemic oxidative stress through biomarkers detected in blood. Such a validation step provides evidence of the clinical potential of OE-biofunctionalized wool as a wound dressing material, bridging the gap between laboratory research and practical medical application.

**Figure 1:**
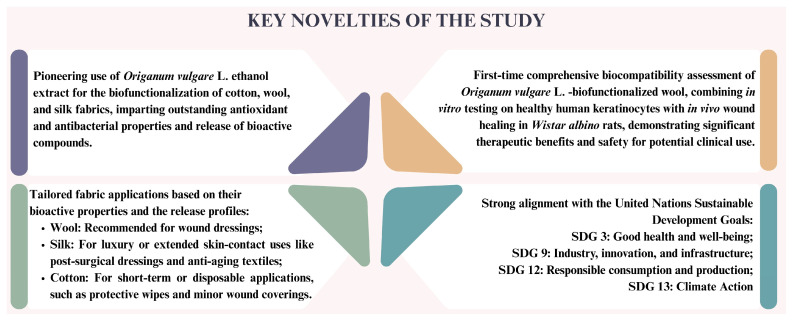
Key novelties of the study.

The research described in the manuscript aimed to confirm the hypotheses: (1) OE can effectively biofunctionalize cotton, wool, and silk fabrics, imparting them with enhanced bioactive properties; (2) OE-biofunctionalized cotton, wool, and silk fabrics will exhibit distinct biocompatibility and release profiles, supporting their potential for tailored applications in healthcare and medical textiles; (3) OE-biofunctionalized wool fabric will promote wound healing and reduce oxidative stress in vivo, thereby confirming its therapeutic potential as a wound dressing material. It should be emphasized that the study introduces several novel aspects that underscore its contribution to the field of healthcare and medical textiles, Figure 1. Moreover, the investigations carried out in this work are aligned with the One Health framework promoted by the World Health Organization (WHO), while concurrently supporting key policy objectives of the European Green Deal and advancing the United Nations Sustainable Development Goals (SDGs), particularly SDGs 3 (Good Health and Well-being), 9 (Industry, Innovation and Infrastructure), 12 (Responsible Consumption and Production), and 13 (Climate Action).

## 2. Materials and Methods

### 2.1. Preparation and Characterization of OE

Bioactive compounds were extracted from *Origanum vulgare* L. using 70% ethanol at a liquid-to-solid ratio of 40 mL/g. The extraction process was carried out at 60 °C for 30 min employing an ultrasonic liquid processor. Subsequently, the resulting OE underwent centrifugation at 6000 rpm for 5 min, followed by a twofold dilution with distilled water.

Qualitative and quantitative analysis of the OE was performed using a Waters ACQUITY Ultra Performance Liquid Chromatography (UPLC) system equipped with a photodiode array (PDA) detector (Milford, MA, USA). Before injection, the original extract was diluted tenfold and filtered through 0.22 µm nylon syringe filters. Data acquisition was managed with Empower version 2 software. Separation was achieved on an ACQUITY UPLC™ BEH C18 column (1.7 µm particle size, 100 mm × 2.1 mm, Waters). The mobile phase consisted of solvent A (10% acetonitrile in water) and solvent B (100% acetonitrile). The flow rate was set to 0.2 mL/min, with an injection volume of 3 µL. The elution program was as follows: 5% to 55% B from 0 to 12 min, returned to 5% B from 12 to 13 min, and held at 5% B until 15 min. UV-Vis spectra were collected in the 200–500 nm range, with chromatograms recorded at 280 nm using the PDA detector. The components were identified by comparing their retention times and UV-Vis spectra with the corresponding analytical standards.

The antioxidant activity of the prepared extract was evaluated using the ABTS radical scavenging assay, as described by Pavun et al. [11]. Measurements were performed spectrophotometrically, and the results represent the mean of three independent determinations. The coefficient of variation across replicates was less than 2.1%.

The antibacterial activity of the OE was assessed against the Gram-negative bacterium *E. coli* ATCC 25922 and the Gram-positive bacterium *S. aureus* ATCC 25923, following the protocol described by Ivanovska et al. [12]. All experiments were performed in triplicate, with the coefficient of variation maintained below 1.7%.

### 2.2. OE-Biofunctionalization of Cotton, Wool, and Silk Fabrics and Their Characterization

Diluted OE was employed for the biofunctionalization of commercially produced natural fabrics, including cotton (CO), wool (WO), and silk (SILK). For each treatment, 1 g of fabric was immersed in 25 mL of OE and shaken in a water bath at 40 °C for 24 h. OE-biofunctionalized fabrics were rinsed with distilled water and air-dried at room temperature.

The antioxidant activity of the OE-biofunctionalized fabrics was assessed using the ABTS radical scavenging assay, following the procedure described by Pavun et al. [12]. Results are expressed as the mean of three independent measurements, with a coefficient of variation below 2.1%.

Antibacterial activity of OE-biofunctionalized fabrics was evaluated against the Gram-negative bacterium *Escherichia coli* ATCC 25922 and the Gram-positive bacterium *Staphylococcus aureus* ATCC 25923, in accordance with the ASTM E2149-01 (2001) standard test method. The results represent the average of three independent replicates, with a coefficient of variation below 3.4%.

To investigate the release of bioactive compounds from the OE-biofunctionalized fabrics, samples were immersed in a physiological saline solution (9 g/L NaCl) and maintained at 37 °C. The release of bioactive compounds was monitored using UV-Vis spectroscopy at predefined time intervals: 1 h, 3 h, 5 h, 24 h, and 48 h.

WO and WO+OE fabric surface chemistry was assessed using ATR-FTIR spectroscopy (Nicolet™ iS™ 10 FT-IR, Thermo Fisher 2 SCIENTIFIC, Waltham, MA, USA), while FTIR spectra were recorded in the range of 4000–400 cm^−1^ with 32 scans per spectrum.

Field Emission Scanning Electron Microscopy (FESEM, Tescan MIRA 3 XMU, Brno, Czech Republic) was used to evaluate changes on fabrics’ surfaces. Before the FESEM analysis, all samples were sputter-coated with a thin layer of gold.

The cytotoxicity of the WO+OE was tested on human keratinocyte cells (HaCaT cell line obtained from ATCC), in accordance with a previously described method [13]. The MTT assay was performed in quadruplicate and results were expressed as percentages relative to the control (untreated cells), which were arbitrarily set to 100%. The cell viability (%) was calculated as follows:

Cell viability (%) = (A of the treated group/A of the control group) × 100(1)

### 2.3. In Vivo Wound Healing Study

This part of the study was conducted at the Center of Excellence for Redox Balance Research in Cardiovascular and Metabolic Disorders, Faculty of Medical Sciences, University of Kragujevac, Republic of Serbia. The experimental protocol was approved by the Ethics Committee for the Welfare of Experimental Animals of the same institution (Approval No. 01–6292). All procedures complied with the European Union Directive 86/609/EEC on the protection of animals used for scientific purposes and adhered to the highest ethical standards.

#### 2.3.1. Animals and Excision Wound Model

A total of 40 male *Wistar* albino rats (*Rattus norvegicus*, *Wistar* strain, body weight 220 ± 30 g, 8–10 weeks old) were procured from the Military Medical Academy, Belgrade, Republic of Serbia. Animals were housed in standard cages (a single animal per cage) under controlled environmental conditions (12/12 h light/dark cycle, 22 ± 2 °C), with free access to standard chow and water ad libitum. Animals had not been subjected to any prior experimental procedures and underwent a 7-day acclimatization period to the housing environment before the study. All animals were of normal immune status, with no genetic modifications or specific genotypes.

Before wound induction, rats were anesthetized via intraperitoneal injection of a ketamine (5 mg/kg) and xylazine (10 mg/kg) mixture. The dorsal surface was shaved and disinfected with 70% ethanol. A full-thickness excision wound (1 cm × 1 cm) was created using a sterile scalpel, extending through the epidermal, dermal, and partial hypodermal layers, as described by Gul Satar et al. [14]. Post-surgery, rats were housed individually and randomly allocated into four experimental groups using pre-existing tables of random numbers to ensure unbiased group assignment:

- NC (negative control): wounds left untreated;
- PC (positive control): wounds treated daily with 0.5 g of 1% silver sulfadiazine cream;
- WO (wool fabric): wounds treated daily with wool fabric, with dressing replaced daily;
- WO+OE (OE-biofunctionalized wool): wounds treated with OE-biofunctionalized wool fabric, with dressing replaced daily.

The respective treatments were applied once daily for 14 consecutive days and potential confounders such as the order of treatments and measurements, as well as animal cage locations, were minimized by randomizing treatment order, measurement sequence, and cage placement. Five animals per group were euthanized on days 7 and 14 for subsequent analyses. Euthanasia was performed under short-term ketamine/xylazine anesthesia followed by decapitation. There were no exclusions among the experimental rats, as none exhibited post-surgical mortality, significant morbidity, infection, or abnormal behavior that would necessitate removal. All rats that completed the experimental protocol were included in the final analysis; for each analysis, 5 animals from day 7 and 5 animals from day 14 post-treatment were included. The primary outcome measures assessed in this study included wound contraction, quantification of hydroxyproline content and evaluation of oxidative stress markers in blood and tissue samples. Group allocation and treatment administration were performed by one researcher, while outcome assessments and data analysis were conducted independently by a second researcher who was blinded to group assignments, thereby ensuring unbiased evaluation of the experimental results. No unexpected adverse events were observed during the study. 

Animals were closely monitored daily throughout the study for any signs of pain, distress, or abnormal behavior, including changes in activity, grooming, posture, and food or water intake. Any unexpected adverse effects or signs of suffering would have prompted immediate intervention, including administration of additional analgesics or humane euthanasia if necessary, to ensure animal welfare was maintained at all times. Nevertheless, all animals tolerated the procedures well, with no signs of excessive pain, infection, or complications related to the wound or treatment.

#### 2.3.2. Wound Healing Rate and Biochemical Analyses

Wound areas were documented and photographed on days 0, 7, and 14 after wound creation using graph paper and analyzed with ImageJ software v 1.54. The wound healing rate was calculated on days 7 and 14 using the following equation:

Wound healing rate (%) = (Wound area on day 0−Wound area on particular day)/Wound area on 0 day(2)

On days 7 and 14, skin tissue and blood samples were collected for biochemical analysis. Skin samples were analyzed for hydroxyproline content using the method described by Andjić et al. [15]. Additionally, tissue redox status was assessed by measuring superoxide dismutase (SOD), catalase (CAT), glutathione (GSH), and index of lipid peroxidation (determined as TBARS), in tissue lysates as described by Patro et al. [16].

Blood samples were collected from the jugular vein to evaluate systemic redox status. Antioxidant parameters (SOD, CAT, and GSH) were analyzed in erythrocyte lysates, while plasma samples were evaluated for pro-oxidative markers including hydrogen peroxide (H_2_O_2_), superoxide anion radical (O_2_^−^), nitrite ion (NO_2_^−^), and the index of lipid peroxidation (measured as TBARS), following the methodology of Andjić et al. [15].

#### 2.3.3. Statistical Analysis

A sample size of 10 rats per group was calculated using G*Power 3 software, version 3.1.3.9 (Heimrich-Heine-Universität, Düsseldorf, Germany) based on previously published data employing a similar experimental design with wound contraction as the primary parameter for sample size determination [17]. Statistical analyses were performed using IBM SPSS Statistics software, version 23.0 (IBM Corp., Armonik, NY, USA). Data distribution was assessed using the Shapiro–Wilk test. Depending on the distribution, either parametric tests (one-way ANOVA, independent-samples *t*-test) or non-parametric tests (Kruskal–Wallis) were applied to evaluate intergroup differences. A *p*-value < 0.05 was considered statistically significant. Data are presented as mean ± standard deviation (SD). Comparisons between groups at each time point were performed using one-way ANOVA, followed by Tukey’s post hoc test. Comparisons between day 7 and day 14 within the same group were conducted using a two-tailed Student’s *t*-test.

## 3. Results and Discussion

### 3.1. Chemical Profiling and Bioactivity of OE

Despite the abundance of recent literature dealing with the chemical profiling of OE [18,19,20], variations in plant geographical origin, environmental conditions, and extraction methods exert substantial influence on its chemical profile and, consequently, its bioactivity. Therefore, chemical profiling using UPLC was necessary to validate the presence of key bioactive compounds and evaluate the extract’s suitability for healthcare-oriented applications. As summarized in Table 1, rosmarinic acid was the most abundant compound in studied OE, with a concentration of 137.00 µg/mL, followed by catechin, which was also present at a relatively high concentration, 77.34 µg/mL. Additional compounds identified in the OE include hydroquinone, isoorientin, and rutin. The presence of phenolic acids such as gallic, caffeic, and salicylic acids, alongside flavonoids such as rutin and isoorientin, further corroborates the extract’s substantial polyphenolic profile. Besides polyphenols, the monoterpenoids carvacrol and thymol were also identified, albeit at lower concentrations compared to other phytochemicals, Table 1. Overall, the chemical profiling of OE presented in Table 1 aligns with previously published data [21,22,23].

Compounds identified in OE are well-documented for their diverse biological activities, including antioxidant, antimicrobial, and anti-inflammatory properties [21,24,25], as well as their potential health-promoting effects. As anticipated, the mixture of phytochemicals acts synergistically, resulting in 100% antioxidant activity of OE, as assessed by the ABTS test. The high antioxidant activity of OE can be primarily attributed to the abundance of rosmarinic acid, catechin, and rutin, which are known to scavenge free radicals and inhibit oxidative stress pathways effectively [12,26,27,28]. In addition to its potent antioxidant properties, OE demonstrated remarkable antibacterial efficacy, achieving a 99.99% reduction of both tested pathogenic strains, *E. coli* and *S. aureus*. The strong antimicrobial activity is likely due to the presence of both phenolic acids and monoterpenoids [29,30,31,32], which are recognized for their ability to disrupt bacterial cell membranes, interfere with intracellular metabolic processes, and inhibit biofilm formation. These findings underscore the potential of OE as a valuable source of bioactive compounds, supporting its application in developing healthcare and medical textiles.

Although the present study primarily focused on the development of natural fabrics biofunctionalized with *Origanum vulgare* L. extract, the assessment of dermal absorption and potential systemic exposure is essential for evaluating its long-term safety in healthcare and medical applications. Evidence from the literature indicates that the primary bioactive compounds of OE exhibit limited dermal absorption and are considered safe, even in cases of systemic exposure. Ex vivo studies using human skin have demonstrated that rosmarinic acid predominantly accumulates in the epidermis, with negligible permeation into the dermis and almost undetectable levels in systemic circulation [33]. Moreover, rosmarinic acid possesses inherently low bioavailability and is rapidly metabolized into conjugated and methylated forms, which are excreted via urine, thereby reducing the risk of systemic accumulation [34]. Similarly, thymol and catechin, both classified as Generally Recognized as Safe (GRAS) by the U.S. Food and Drug Administration, exhibit limited dermal absorption, predominantly remaining within the stratum corneum and viable epidermis [35,36,37,38]. Rutin has also been detected in trace amounts within skin layers, yet exhibits low transdermal permeation, with no statistically significant differences in concentration between the epidermal and dermal layers [38]. These findings suggest that systemic bioavailability of OE compounds via dermal contact is likely minimal, particularly when incorporated into textiles. Nevertheless, further in vivo studies are warranted to more comprehensively assess the potential effects of long-term exposure under real-world conditions.

### 3.2. OE-Biofunctionalized Natural Fabrics

Commercially produced cotton (CO), wool (WO), and silk (SILK) fabrics were biofunctionalized with OE, the chemical composition and bioactivity of which were previously confirmed. The fabrics were immersed in OE and subjected to agitation in a water bath at 40 °C for 24 h to facilitate maximal uptake of the extract’s bioactive compounds. Subsequently, the bioactivity, i.e., antioxidant and antibacterial activities of both raw and biofunctionalized fabrics, was evaluated and compared.

**Figure 2:**
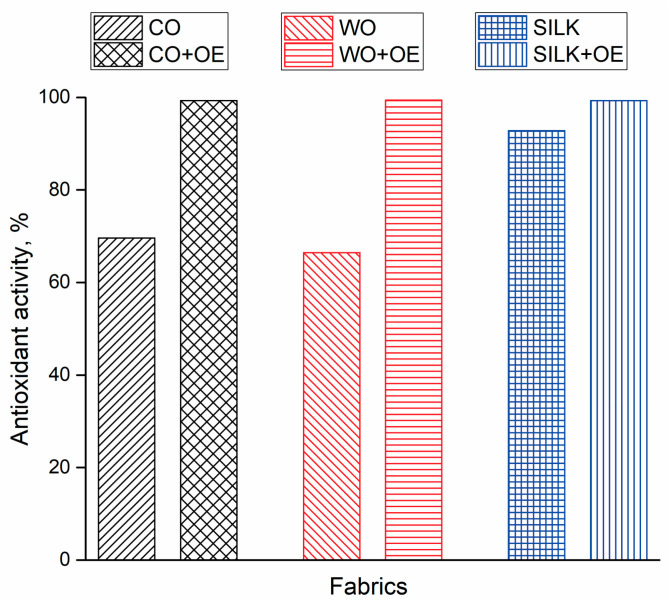
Antioxidant activity of raw and OE-biofunctionalized fabrics.

Studying the antioxidant activity of fabrics intended for healthcare and medical applications is essential since oxidative stress caused by elevated levels of reactive oxygen species (ROS) plays a significant role in the pathogenesis of skin disorders, infections, impaired wound healing, and can lead to tissue damage and cell death [39]. Therefore, the radical scavenging activity of raw and OE-biofunctionalized fabrics was determined and the obtained results are presented in Figure 2. CO, WO, and SILK fabrics demonstrated antioxidant activities of 69.6%, 66.4%, and 92.8%, respectively. Upon biofunctionalization with OE, both CO+OE and WO+OE showed a marked increase in radical scavenging activity, reaching levels comparable to the pure OE (>99% inhibition). The outstanding antioxidant activity of all biofunctionalized fabrics can be attributed to the presence and favorable orientation of functional groups (primarily hydroxyl groups) from potent antioxidant compounds in extract [40], such as rosmarinic acid, catechin, and rutin. The results of this section suggest that SILK fabric, even in its raw form, along with all OE-biofunctionalized fabrics, possesses the potential to mitigate oxidative processes, thereby reducing the likelihood of cellular dysfunction and oxidative damage [41].

It is commonly known that natural fibers like cotton, wool, and silk provide a favorable environment for microbial growth due to their good moisture retention ability. This limitation can be effectively addressed through fiber functionalization with natural antimicrobial agents [12,40,42]. Accordingly, a primary aim of this study was to develop natural fabrics that not only exhibit strong antioxidant activity but also demonstrate outstanding antibacterial activity, an essential attribute for preventing skin infections. Two of the most commonly studied and clinically relevant pathogens, *E. coli* and *S. aureus*, were selected to evaluate antibacterial efficacy. These bacteria are frequently implicated in wound infections, particularly in surgical wounds, which are especially vulnerable to microbial colonization [43]. The results listed in Table 2 indicate that CO exhibited inherent antibacterial activity against *S. aureus* but was ineffective against *E. coli*. After biofunctionalization with OE, the antibacterial performance of cotton was enhanced, with CO+OE achieving 54.11% inhibition against *E. coli* and 60.27% against *S. aureus*. The other two tested natural fabrics, WO and SILK, displayed no antibacterial activity against either tested strain. However, their OE-biofunctionalized counterparts, WO+OE and SILK+OE, showed exceptional antibacterial properties, with WO+OE achieving complete inhibition of both bacteria, and SILK+OE showing strong antibacterial activity exceeding 89%, Table 2. These results suggest a high affinity between OE bioactive compounds and protein-based fibers, which likely facilitates greater compound bonding and retention, leading to excellent antimicrobial action. Collectively, the findings validate the OE-biofunctionalization approach as an effective strategy for conferring dual antioxidant and antibacterial properties to natural textiles.

**Figure 3:**
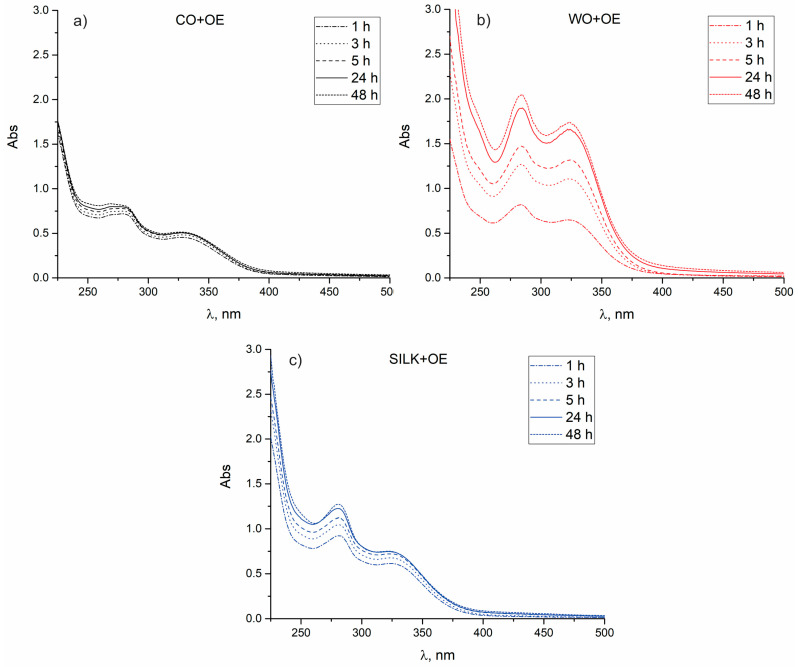
Time-dependent release of OE from: (**a**) CO+OE, (**b**) WO+OE, and (**c**) SILK+OE fabrics.

Given that the main objective of this study was to evaluate the suitability of OE-biofunctionalized fabrics for various healthcare and medical applications, besides confirming their bioactivity, studying their ability to release OE-derived bioactive compounds is crucial [26]. Therefore, UV-Vis spectroscopy was employed to investigate the time-dependent release of OE from CO+OE, WO+OE, and SILK+OE fabrics. The in vitro experiments were performed over 48 h at 37 °C in a physiological saline solution, mimicking the body fluids. The results presented in Figure 3a revealed that among the studied fabrics, CO+OE demonstrated the lowest overall release efficiency, reaching equilibrium within 3 h. Compared to CO+OE, WO+OE showed a remarkably different release profile. Specifically, the absorbance values were significantly higher, particularly in the early hours (1 h to 5 h), implying a more pronounced release of OE bioactive compounds from WO+OE, as depicted in Figure 3b. However, between 24 h and 48 h, the rate of absorbance increase diminished, suggesting a slower release. The third tested OE-biofunctionalized fabric, SILK+OE, displayed an intermediate release of OE bioactive compounds, with a longer-lasting release than CO+OE but lower than WO+OE. The obtained results pointed out that after an initial rapid release within the first 5 h, equilibrium was approached at 24 h, Figure 3c.

Each fabric’s unique bioactivity and release profile can be tailored for specific healthcare applications. Specifically, CO+OE is well-suited for protective textiles intended for minor cuts and abrasions, temporary bandages, or disposable bioactive medical wipes, where strong antibacterial activity and prolonged bioactive compound release are not required. SILK+OE, which provides excellent bioactivity and a moderate yet prolonged release of bioactive compounds, is particularly suitable for applications that demand both comfort and extended therapeutic benefits. These include luxury therapeutic fabrics, skin-care textiles, post-surgical dressings, and anti-aging textiles. Finally, WO+OE, with its sustained release profile and excellent bioactivity, is ideal for wound dressings, diabetic socks, antibacterial hospital linens, and therapeutic fabrics designed for applications requiring continuous antioxidant and antimicrobial effects.

### 3.3. Testing the Suitability of WO+OE for Application as Wound Dressing

#### 3.3.1. Surface and Chemical Characterization of WO and WO+OE: Insights into OE–Wool Interactions

**Figure 4:**
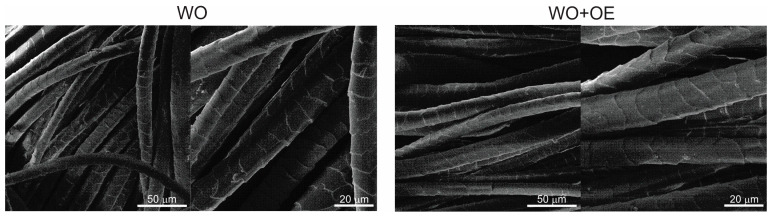
Microphotographs of WO and WO+OE.

The SEM micrographs reveal that both WO and WO+OE exhibit the characteristic scaly surface structure typical of wool, Figure 4. Importantly, no apparent morphological alterations are observed upon OE-biofunctionalization; the scaly architecture remains intact in the WO+OE, with no signs of surface damage, roughening, or film formation. This confirms that the OE-biofunctionalization process does not compromise the structural integrity of the wool. As expected, the functionalization with OE occurs at the molecular level, preserving the wool fiber’s native surface morphology.

**Figure 5:**
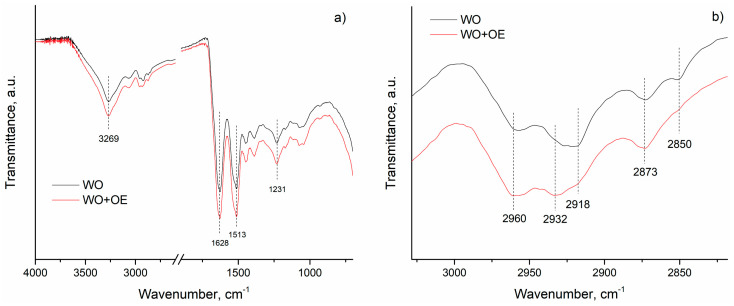
ATR-FTIR spectra of WO and WO+OE in the range of: (**a**) 4000–700 cm^−1^ and (**b**) 3020–2810 cm^−1^.

To elucidate the interactions responsible for the adsorption of OE’s bioactive compounds onto WO fabric, ATR-FTIR spectra of WO and WO+OE were compared, Figure 5. The characteristic band at 3269 cm^−1^ corresponds to N–H stretching vibrations of amide A, while the band at 1628 cm^−1^ is attributed to C=O stretching vibrations of amide I, Figure 5a. The band observed at 1513 cm^−1^ is assigned to the combination of N–H in-plane bending and C–N stretching vibrations associated with the protein backbone (amide II) in WO. Furthermore, the peak at 1231 cm^−1^, corresponding to amide III, arises from C–N and C–O stretching vibrations. All characteristic bands of WO are observed in the WO+OE spectrum, with a slight increase in intensity, indicating preservation of the primary protein structure after the OE-biofunctionalization. The most prominent spectral changes between WO and WO+OE are observed in the region corresponding to aromatic and aliphatic C–H stretching vibrations (3000–2800 cm^−1^), Figure 5b. In particular, the band at 2932 cm^−1^ becomes more intense, whereas the bands at 2918 and 2850 cm^−1^ show reduced intensity upon OE-biofunctionalization. These spectral changes suggest the involvement of aromatic and aliphatic –CH groups, likely originating from amino acid side chains, in the binding of OE-derived compounds.

**Figure 6:**
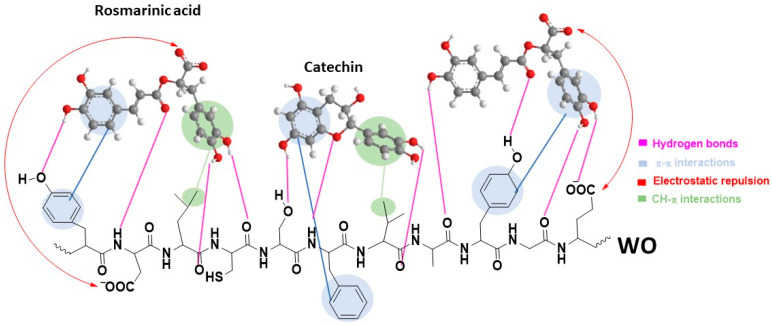
Possible interactions between WO fiber and rosmarinic acid and catechin, as representative compounds of OE.

Given the structural complexity of both the WO and bioactive compounds in OE (Table 1), the binding mechanism is expected to be intricate, governed by multiple types of interactions. Therefore, for simplification, this discussion primarily focuses on rosmarinic acid and catechin, the main compounds in OE, Table 1. Other compounds present in OE share structural similarities with these two compounds. When evaluating potential interactions between these compounds and the WO surface, it is crucial to consider both the nature and types of functional groups present in them, as ionization states, which play a key role in determining the type and strength of interactions. The pH of the OE used for fabric biofunctionalization is 5.9. At this pH, the WO fabric surface carries a net negative charge, as its isoelectric point is estimated at 4.8 [12]. Rosmarinic acid has a p*K*_a_ value of 3.57 [44], indicating that its carboxylic group is deprotonated and the molecule is negatively charged at pH 5.9. Based on these values, it can be assumed that electrostatic repulsion between rosmarinic acid and the negatively charged WO surface likely influences the orientation of the adsorbed molecules, Figure 6. This orientation promotes the formation of hydrogen bonds between the rosmarinic acid –OH groups and hydrogen bond acceptors on WO, as well as between the carbonyl group of rosmarinic acid and hydrogen bond acceptor groups on WO. Additionally, the two phenyl rings in rosmarinic acid contribute to its binding affinity via π–π stacking interactions with aromatic amino acid residues, and CH–π interactions with methyl groups of the aliphatic side chains in wool [45], as evidenced by the FTIR spectra, Figure 5. Catechin, on the other hand, has a p*K*_a_ value above 8.6 [46], and thus remains predominantly in its neutral form at pH 5.9. As such, it is not subject to electrostatic repulsion from the negatively charged WO surface. Similar to rosmarinic acid, catechin adsorption is mainly driven by a combination of hydrogen bonding, π–π stacking, and CH–π interactions, Figure 6.

#### 3.3.2. Cytotoxicity of WO+OE

**Figure 7:**
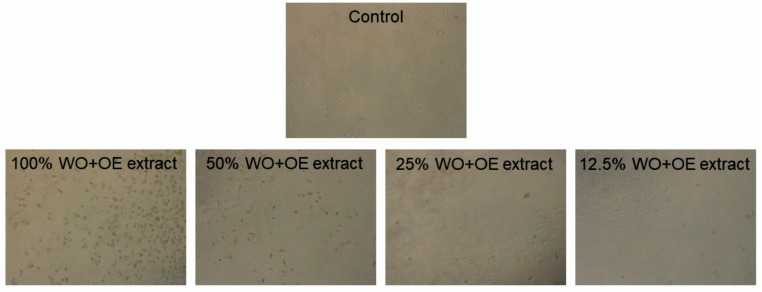
Microphotographs of HaCaT cells in contact with WO+OE extracts of various concentrations.

Given the selection of WO+OE as the most suitable material for wound dressing, and its intended prolonged contact with human skin, assessing its cytotoxicity to healthy skin cells is essential to validate its biocompatibility and overall suitability. Specifically, if a fabric is cytotoxic, it may lead to cell death (apoptosis or necrosis), causing delayed wound healing or infection risk [47]. Human skin comprises keratinocytes, melanocytes, and fibroblasts, with keratinocytes predominating in the outer layer. The in vitro cytotoxicity of WO+OE was tested using a healthy human keratinocyte cell line (HaCaT). WO+OE extracts were prepared by immersing the fabric in RPMI medium under dynamic conditions. The optical microphotographs depicting the viability of HaCaT cells after 48 h of exposure to WO+OE extracts are presented in Figure 7. Significant reduction in cell viability is observed upon exposure to fabric extracts, particularly notable with the 25% and 12.5% WO+OE extract.

**Figure 8:**
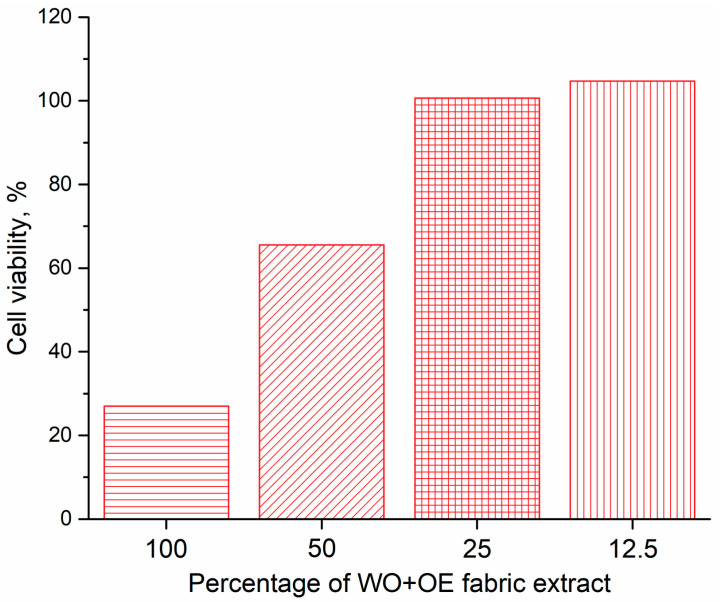
Cytotoxicity of WO+OE fabric extract towards HaCaT.

The previous observation is further corroborated by the MTT assay, used to assess cell viability upon exposure to fabric extracts of varying concentrations, as shown in Figure 8. According to ISO 10993-5 standard, cell viability exceeding 80% indicates non-cytotoxicity, while viability between 60 and 80%, 40 and 60%, and below 40% suggests weak, moderate, and strong cytotoxicity, respectively. As evident from Figure 8, the highest concentration of fabric extract exhibited cytotoxic effects on HaCaT cells, whereas a 50% dilution resulted in approximately 65% cell survival. Interestingly, WO+OE extracts at 25% and 12.5% concentrations promoted cell viability exceeding 100%, indicating non-cytotoxicity under the tested conditions. Considering the 48 h exposure period for cytotoxicity assessment, it is plausible that shorter contact durations, such as 24 h, as is determined the most appropriate duration of treatment with WO+OE wound dressing (Figure 3b), may further confirm its non-toxic nature to HaCaT cells.

It seems this is the right place to stress that the in vitro cytotoxicity experiments were conducted using keratinocytes, which represent a key but single layer of the skin. The unexpected results obtained for cell viability of 100% WO+OE fabric extract suggested the need for further evaluation to better understand the fabric’s biological response across the full complexity of the skin. Human skin is a multi-layered organ composed of the epidermis, dermis, and hypodermis, each with distinct structural and functional characteristics that influence how wound dressing interacts with cells and tissues. As such, in vitro cytotoxicity results obtained from a single cell type, in this case, keratinocytes, may not fully capture the WO+OE behavior in a physiological context. For this reason, and in recognition of the limitations of in vitro cytotoxicity testing, comprehensive in vivo experiments using *Wistar albino* rats were conducted.

#### 3.3.3. In Vivo Evaluation of WO+OE as Wound Dressing

To assess the biocompatibility and therapeutic potential of WO+OE as a wound dressing, in vivo experiments were conducted on *Wistar albino* rats using an excision wound model in addition to prior in vitro cytotoxicity testing on HaCaT cells. For that purpose, the rats were divided into four groups: a negative control group (NC) with untreated wounds, a positive control group (PC) treated daily with 1% silver sulfadiazine cream, and two groups treated with WO or WO+OE, with dressings replaced daily. The progression of wound healing was monitored over 14 days, with particular attention to the influence of treatment type and duration.

**Figure 9:**
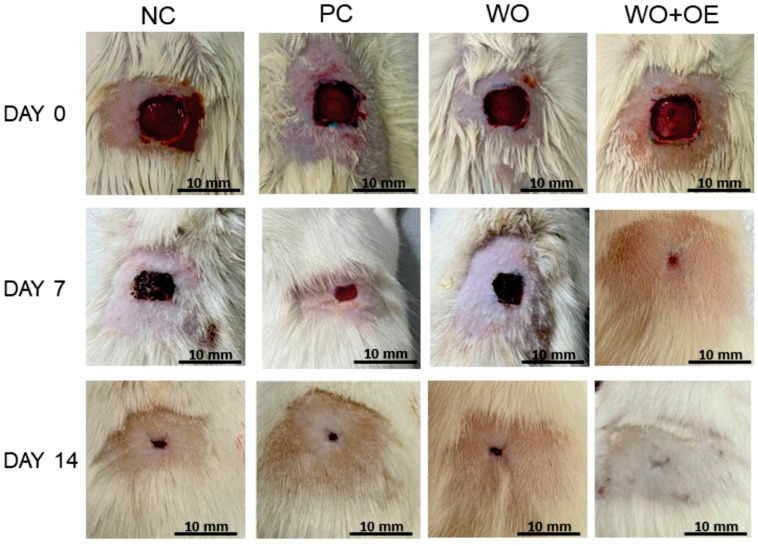
Impact of various treatments on wound contraction.

**Figure 10:**
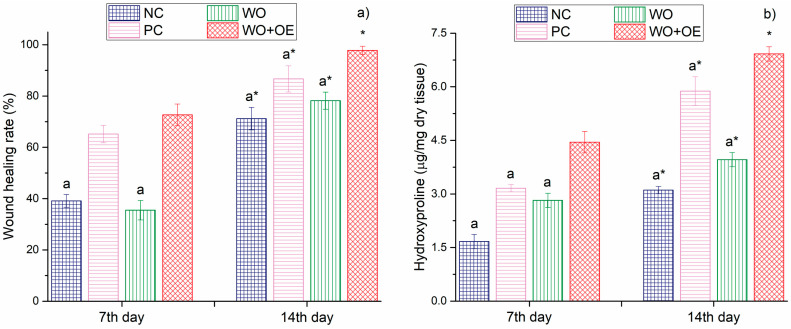
(**a**) Wound healing rate and (**b**) hydroxyproline content among different experimental groups; a—statistical significance relative to the WO+OE group, *—statistical significance relative to day 7.

Visual analysis of wound healing over time revealed progressive healing across all groups, with notable differences in the rate and extent of wound contraction, Figure 9. By day 7, the NC and WO groups exhibited low healing rates of 39.1%, and 35.5%, respectively, Figure 10a. In contrast, the PC group demonstrated a 65.2% healing rate, which is consistent with the known antimicrobial and anti-inflammatory properties of administrated silver sulfadiazine cream [48]. As expected, the WO+OE group showed the most pronounced healing, with a 72.7% reduction in wound size and healthier surrounding skin, suggesting an early pro-healing effect due to the release of OE’s bioactive compounds. Namely, the enhanced wound healing efficacy observed in the rats treated with WO+OE may be attributed to the synergistic action of OE’s phenolic and terpenoid constituents. Rosmarinic acid, the predominant compound in OE (Table 1), exerts potent anti-inflammatory effects by down-regulating pro-inflammatory cytokines such as TNF-α, IL-1β, and IL-6, and by inhibiting the activation of the NF-κB signaling pathway [34,49]. It also suppresses the expression of key inflammatory enzymes including cyclooxygenase-2 (COX-2) and inducible nitric oxide synthase (iNOS) [50]. In addition, rosmarinic acid displays strong antioxidant activity by scavenging reactive oxygen species (ROS) and upregulating endogenous antioxidant enzymes such as superoxide dismutase and catalase, thereby reducing oxidative stress in the wound microenvironment [51,52]. Thymol and carvacrol, through their membrane-disrupting and anti-inflammatory actions, can reduce microbial burden and local inflammation [53,54,55,56,57,58]. These compounds have been reported to promote fibroblast proliferation, keratinocyte migration, and angiogenesis, all of which are critical for tissue regeneration and wound closure [59,60]. These combined pharmacological actions likely contribute to the superior healing outcomes observed in the case of rats treated with WO+OE fabric compared to the PC group of rats treated with standard silver sulfadiazine. The results obtained for wound healing correlate well with hydroxylproline content—a widely recognized marker of collagen deposition and tissue remodeling during wound healing [61], Figure 10b. Precisely, on day 7 post excision, the WO+OE group displayed a significantly elevated hydroxyproline content in skin tissue homogenates (4.45 µg/mg dry tissue) compared to the NC, WO, and PC groups (1.67–3.16 µg/mg dry tissue). This increase is likely attributed to the enhanced recruitment of fibroblasts and epithelial cells to the wound area, thereby promoting early tissue regeneration [62].

By day 14, all rat groups exhibited a marked improvement in wound healing compared to day 7; nevertheless, noticeable variations in wound closure remained. The NC, PC, and WO groups retained visible wound marks, suggesting incomplete healing, Figure 9. Surprisingly, on day 14, the WO+OE group displayed nearly undetectable wound marks, and a statistically significant superior healing rate of 97.8% compared to the other three groups, indicating enhanced tissue regeneration, Figure 9 and Figure 10a. Hydroxyproline analysis supported these findings: in the dry tissue of the WO+OE group, 6.92 µg/mg hydroxyproline was measured, which significantly exceeded the NC, PC, and WO groups (3.11, 5.88, and 3.96 µg/mg dry tissue, respectively), Figure 10b. The enhanced collagen biosynthesis observed in the WO+OE group reflects a positive outcome of the synergistic interaction between the WO and OE’s bioactive compounds, which promote more effective cell proliferation and extracellular matrix formation [63]. This synergism results in accelerated and more efficient wound healing compared to the other treatment groups. Costa et al. [60], Lee et al. [64], Xiong et al. [65], Yin et al. [66], and Zheng et al. [67] reported that rosmarinic acid, catechin, thymol, and carvacrol, which are present in the OE used (Table 1), are well known for their wound healing properties, exerting effects across all phases of healing by modulating inflammation and oxidative stress, as well as promoting tissue regeneration. The strong positive correlation (r = 0.985) observed between hydroxyproline content and wound healing rate at day 14 highlights the role of OE in promoting not only accelerated wound closure but also structurally advanced healing.

The previously discussed wound healing relies on oxidative stress since overproduction of ROS can cause sustained inflammation, cellular damage, and dysfunction, which ultimately hinder tissue repair [68]. Therefore, evaluating tissue and redox status is essential to understand the cellular changes following different treatment types influencing wound repair progression and to assess their therapeutic efficacy. Markers of tissue redox status, including antioxidants superoxide dismutase (SOD), catalase (CAT), glutathione (GSH), and pro-oxidant thiobarbituric acid reactive substance (TBARS), were evaluated as key indicators of local oxidative stress and antioxidant defense mechanisms influencing cellular processes critical to wound healing [69]. SOD is an antioxidant enzyme that catalyzes the dismutation of superoxide radicals into hydrogen peroxide and molecular oxygen [70], while CAT subsequently decomposes hydrogen peroxide into water and oxygen [71], thereby limiting potential oxidative damage and preserving cellular function. GSH functions as a major non-enzymatic antioxidant, protecting cells from oxidative injury through redox buffering and detoxification [72]. In contrast, TBARS serves as a marker of lipid peroxidation, with elevated levels reflecting increased oxidative damage within tissues, which can hinder wound repair.

**Figure 11:**
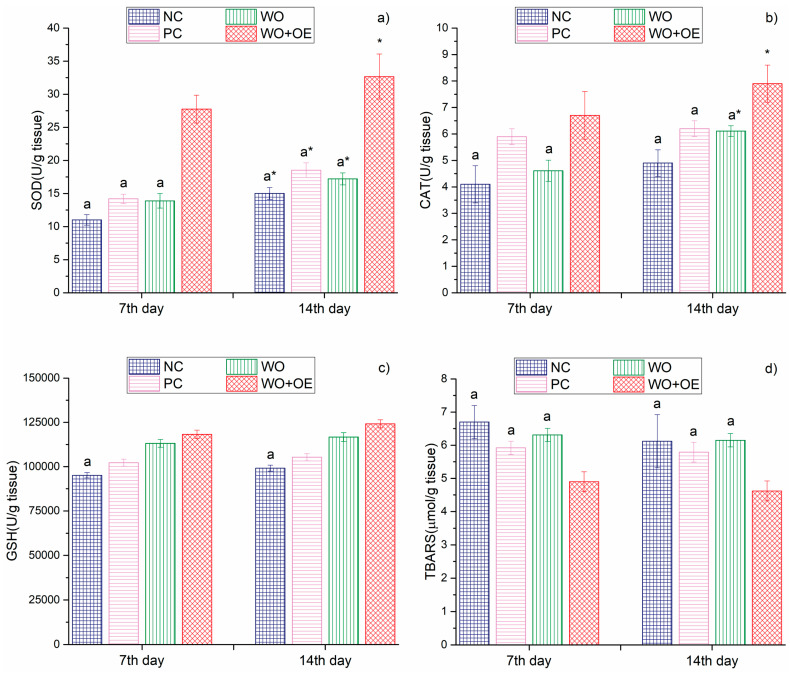
Tissue redox status parameters: (**a**) SOD, (**b**) CAT, (**c**) GSH, and (**d**) TBARS of NC, PC, WO, and WO+OE rat groups. a—statistical significance relative to the WO+OE group, *—statistical significance relative to day 7.

The findings depicted in Figure 11a indicate that, on both the 7th and 14th days, the WO+OE group exhibited significantly elevated SOD levels (27.8 and 32.7 U/g tissue, respectively) compared to NC, PC, and WO groups (11.0–14.2 and 15.0–18.5 U/g tissue, respectively). This behavior suggests that the release of bioactive compounds from OE considerably enhances SOD-mediated dismutation of superoxide radicals, a critical step in oxidative stress mitigation. A similar trend was observed for CAT; on day 14, the WO+OE group demonstrated 21.5–38.0% higher CAT levels in relation to NC, PC, and WO groups, Figure 11b. It is noteworthy that although the differences between GSH levels among the four experimental groups of rats were not statistically significant (Figure 11c), the elevated GSH levels mean enhanced cellular redox buffering capacity and detoxification potential [72] in the WO+OE group. Furthermore, the levels of TBARS are the lowest in the WO+OE group of rats at both time points (4.9 and 4.6 U/g tissue on the 7th and 14th days, respectively), indicating the strong potential of the WO+OE to suppress lipid peroxidation and mitigate wound-induced oxidative damage more effectively than all other treatment groups, Figure 11d. In contrast, the NC, PC, and WO groups maintained relatively higher TBARS levels ranging between 5.9 and 6.7 µmol/g tissue and between 5.8 and 6.2 µmol/g tissue on days 7 and 14, respectively. The discussed data undoubtedly pointed out that the WO+OE group of rats is characterized by statistically significant enhancements in the antioxidant defense system in wound tissues, characterized by increased enzymatic antioxidant levels and reduced lipid peroxidation, which correlated with the most rapid wound healing rate compared to all other groups of rats, Figure 10. The pronounced antioxidant response of WO+OE can be explained by the presence of rosmarinic acid, catechin, carvacrol, and thymol in OE, which are known to enhance the activity of enzymes like SOD, CAT, and glutathione peroxidase (GPx), and reduce lipid peroxidation, as reported in previous studies [60,73,74].

To obtain a comprehensive understanding of the biochemical environment influencing wound healing, systemic redox status (encompassing both the markers of antioxidant defense system and pro-oxidants) was assessed in blood samples since it reflects the overall oxidative balance of the organism [17]. The markers of the antioxidant defense system, including CAT, SOD, and GSH, were quantified in blood cell lysates. Plasma samples were analyzed for pro-oxidant markers, including hydrogen peroxide (H_2_O_2_), superoxide anion radical (O_2_^−^), nitrite ion (NO_2_^−^), and the index of lipid peroxidation (measured as TBARS).

**Figure 12:**
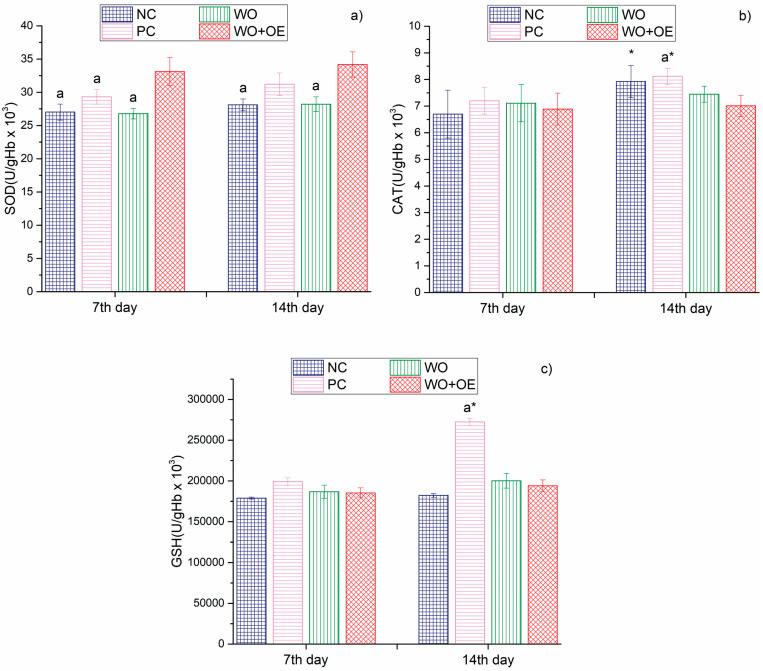
Parameters of antioxidant defense system: (**a**) SOD, (**b**) CAT, (**c**) GSH, of NC, PC, WO, and WO+OE rat groups. a—statistical significance relative to the WO+OE group, *—statistical significance relative to day 7.

The systemic redox status data presented in Figure 12 indicate that the rats’ treatment with WO+OE influenced the organisms’ overall oxidative balance throughout the wound healing period. On both the 7th and 14th days, the WO+OE group exhibited the highest levels of SOD (33.1 and 34.2 U/gHb × 10^3^), in comparison to the other groups, whose values ranged from 26.8 to 31.2 U/gHb × 10^3^, Figure 12a. This statistically significant increase proves that the release of OE from the WO+OE effectively enhances the systemic dismutation of superoxide anion radicals (O_2_^−^) [75]. CAT activity showed no significant differences among the studied groups of rats on day 7 (Figure 12b). This behavior can be ascribed to the possible presence of a tightly regulated hydrogen peroxide detoxification system potentially mediated in part by glutathione peroxidase, which was sufficient to complement the elevated SOD activity [76,77]. However, by day 14, the WO+OE group demonstrated a marked reduction in CAT level relative to the PC group. This reduction in CAT activity may suggest that H_2_O_2_ was potentially detoxified through GPx, which effectively complements elevated SOD activity and maintains redox balance [78]. In contrast, the 1% silver sulfadiazine-treated group of rats (PC) demonstrated a significant rise in CAT activity by day 14, likely as a reaction to enhanced ROS production. The last marker of the antioxidant defense system, GSH, remained relatively consistent across all groups on day 7, Figure 12c. The situation is somewhat different on day 14; the GSH level of the PC group rose sharply, whereas the levels in the WO+OE group remained within the physiological range. The last one means that the antioxidant effect of the WO+OE was primarily mediated through enzymatic pathways rather than through the augmentation of GSH levels. The stable GSH values in the WO+OE group may also indicate less systemic oxidative challenge, consistent with the effective local ROS neutralization observed in wound tissue, Figure 12.

**Figure 13:**
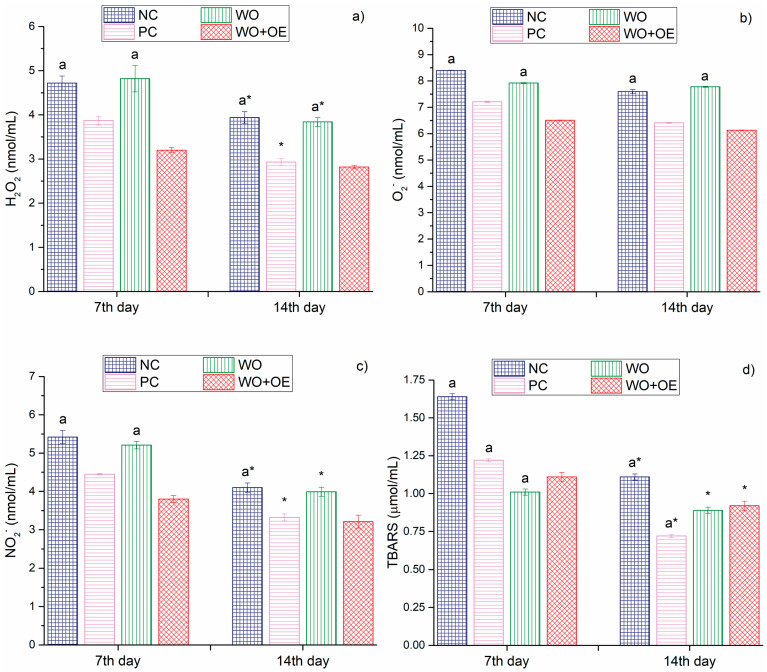
Pro-oxidant markers: (**a**) H_2_O_2_, (**b**) O_2_^–^, (**c**) NO_2_^–^, and (**d**) TBARS of NC, PC, WO, and WO+OE, a—statistical significance relative to the WO+OE group, *—statistical significance relative to day 7.

The last part of the in vivo research involved the evaluation of systemic oxidative burden by quantifying key pro-oxidant markers in plasma samples, Figure 13. Regardless of the time points, day 7 or day 14, H_2_O_2_ and O_2_^–^ levels were significantly lower in rats treated with WO+OE, especially compared to NC and WO, Figure 13a,b. Moreover, the level of NO_2_^–^, a marker of nitric oxide metabolism and oxidative stress, was significantly reduced in the WO+OE group (accounting for 3.8 and 3.2 nmol/mL on 7th and 14th day, respectively) compared to the NC group (accounting for 5.4 and 4.1 nmol/mL on 7th and 14th day, respectively), indicating a dampened nitrosative stress response associated with the antioxidant action of OE’s bioactive compounds [79]. The most pronounced differences in the pro-oxidant markers were observed in TBARS levels, Figure 13c. Namely, on day 7, the TBARS level was the highest in the NC group (1.64 µmol/mL) and significantly lower in the WO+OE group (1.1 µmol/mL). This difference became more evident by day 14, when the TBARS level in the WO+OE group dropped further down to 0.9 µmol/mL. The above discussion clearly demonstrates the capacity of OE to reduce lipid peroxidation during the later phases of wound healing. Overall, the presented data support the hypothesis that the release of bioactive compounds from OE can attenuate systemic oxidative damage, thereby supporting a more favorable biochemical environment for wound repair. The coordinated modulation of antioxidant enzymes SOD and CAT (Figure 12a,b) corresponds well with the observed reductions in systemic pro-oxidants H_2_O_2_ and O_2_^–^ (Figure 13a,b), highlighting an effective balance between ROS production and detoxification that underlies the improved oxidative status and wound healing outcomes in the WO+OE group.

The main conclusion from in vivo experiments is that developed WO+OE demonstrated superior wound dressing performance across all measured parameters since it significantly accelerated wound closure, improved collagen synthesis, enhanced both tissue and systemic antioxidant defenses, and reduced oxidative stress markers in skin tissue and blood samples. It can be postulated that these effects are probably mainly driven by the release of bioactive compounds from OE, making the WO+OE fabric a promising candidate for improving the management of chronic wounds and a promising alternative to commercial antibiotics (like silver sulfadiazine) used in wound care protocols.

Anticipated potential advantages that justify further clinical research of WO+OE are as follows:

(1) Many commercially available wound dressings use synthetic antimicrobial agents (e.g., silver nanoparticles, polyhexanide, etc.). WO+OE provides antioxidant, antibacterial, and anti-inflammatory properties using natural plant-based compounds from OE, potentially minimizing the risk of cytotoxicity, allergic reactions, or microbial resistance.
(2) WO has excellent inherent moisture absorption and desorption properties, helping to maintain an optimal moist environment for wound healing and offering excellent patient comfort.
(3) OE provides both antioxidant activity and antibacterial efficacy against *S. aureus* and *E. coli*, which can work synergistically to promote wound healing by reducing oxidative stress, inflammation, and infection risks, not always addressed simultaneously by commercial dressings.
(4) Both WO and OE are renewable and biodegradable resources, offering an environmentally friendly alternative to synthetic-based wound dressings. Depending on the production scale, using readily available WO and OE could potentially reduce production costs compared to more complex synthetic or nanomaterial-based dressings.
(5) WO+OE is anticipated to significantly reduce both direct and indirect healthcare costs, lower the frequency of hospital admissions, and ultimately support improved clinical outcomes.

Although WO+OE exhibited superior wound healing efficacy compared to silver sulfadiazine, a commercially available antimicrobial agent, comparative studies with additional commercial products are planned for future phases of the research. This investigation was limited to preclinical experiments conducted in an animal model. Future clinical trials and cost-effectiveness evaluations are essential to gain comprehensive insights into the safety, therapeutic efficacy, and economic implications of the proposed treatment in human patients. The clinical translation of WO+OE will require rigorous validation of its safety, stability, and compliance with regulatory standards. Nevertheless, the use of natural fabrics functionalized with naturally derived compounds exhibiting multifunctional bioactivity aligns well with current trends in sustainable and personalized medicine, presenting a promising avenue for future development.

## 4. Conclusions

Here, we report the successful development of bioactive natural textiles, wool, cotton, and silk, with significant potential for various healthcare and medical applications. Specifically, OE-biofunctionalized wool (WO+OE) demonstrates strong antioxidant and antimicrobial properties, alongside a controlled release of OE’s bioactive compounds, positioning it as particularly promising for wound dressing applications. To validate the safety and biocompatibility of WO+OE, we conducted in vitro testing using a healthy human keratinocyte cell line, complemented by in vivo evaluation in *Wistar albino* rats. Animal model studies revealed accelerated wound closure (97.8% by day 14), enhanced collagen synthesis (6.92 µg/mg hydroxyproline content by day 14), enhanced tissue and systemic antioxidant defenses, and reduced oxidative stress markers in skin tissue and blood samples of rats treated with WO+OE. These findings underscore the potential of WO+OE as an advanced solution for managing chronic wounds.

Moreover, applying the same OE-biofunctionalization protocol to cotton and silk (CO+OE and SILK+OE, respectively) fabrics resulted in distinct levels of bioactivity and different release behavior of OE-derived bioactive compounds, highlighting their potential for diverse healthcare applications. For instance, CO+OE is well-suited for short-term or disposable applications, such as protective textiles for minor cuts and abrasions, temporary bandages, or disposable bioactive medical wipes, where strong antibacterial activity and release of bioactive compounds are not essential. Furthermore, SILK+OE exhibits excellent bioactivity with a moderate yet prolonged release profile, making it suitable for applications that require both wear comfort and extended therapeutic effects. These include high-end therapeutic textiles, skin-care fabrics, post-surgical dressings, and anti-aging applications.

The materials developed in this study represent a prototype of bioactive textiles, and are not finalized or clinically approved medical textiles. While the results are promising and suggest potential for future therapeutic application, the bioactive fabrics have not yet been evaluated in accordance with standardized regulatory frameworks. Further research will be necessary to ensure compliance with relevant safety and performance standards, and conduct comprehensive clinical evaluations to support potential clinical translation.

## Figures and Tables

**Table 1:** Chemical composition of OE (RT—retention time).

Compound	RT, Min	Concentration, µg/mL
thymol	12.57	0.45
carvacol	12.20	2.86
rosmarinic acid	5.42	137.00
gallic acid	1.54	4.51
salicylic acid	6.17	0.53
isoorientin	4.02	8.97
rutin	3.90	6.81
hydroquinone	1.80	11.48
catechin	2.63	77.34
caffeic acid	3.25	1.58

**Table 2:** Antibacterial activity of raw and OE-biofunctionalized fabrics.

Fabrics	*E. coli*, %	*S. aureus*, %
CO	/	25.92
CO+OE	54.11	60.27
WO	/	/
WO+OE	99.99	99.99
SILK	/	/
SILK+OE	89.92	91.34

## Data Availability

Data will be made available on request.
